# Growth Performance, Growth-Related Genes, Digestibility, Digestive Enzyme Activity, Immune and Stress Responses of *de novo* Camelina Meal in Diets of Red Seabream (*Pagrus major*)

**DOI:** 10.3390/ani11113118

**Published:** 2021-10-31

**Authors:** Kumbukani Mzengereza, Manabu Ishikawa, Shunsuke Koshio, Saichiro Yokoyama, Zhang Yukun, Ronick S. Shadrack, Seok Seo, Tomonari Kotani, Serge Dossou, Mohammed F. El Basuini, Mahmoud A. O. Dawood

**Affiliations:** 1 The United Graduate School of Agriculture Sciences, Kagoshima University, 1-21-24 Korimoto, Kagoshima 890-0056, Japan; kumbumzenge@gmail.com (K.M.); zhangyukun672@gmail.com (Z.Y.); rspenly@gmail.com (R.S.S.); seoseok91@naver.com (S.S.); 2 Laboratory of Aquatic Animal Nutrition, Faculty of Fisheries, Kagoshima University, Kagoshima 890-0056, Japan; ishikawa@fish.kagoshima-u.ac.jp (M.I.); k9622498@kadai.jp (S.K.); yokoyama@fish.kagoshima-u.ac.jp (S.Y.); 3 Department of Aquatic and Fisheries Science, Private Bag 201, Mzuzu University, Mzuzu 105200, Malawi; 4 Laboratory of Larval Rearing Management, Faculty of Fisheries, Kagoshima University, Kagoshima 890-0056, Japan; kotani@fish.kagoshima-u.ac.jp; 5 Laboratoire d’Hydrobiologie et Aquaculture, Faculté des Sciences Agronomies’, Université d’Abomey Calavi, Cotonou 01 BP:526, Benin; sergedos@yahoo.fr; 6 Faculty of Desert Agriculture, King Salman International University, South Sinai 46612, Egypt; m_fouad_islam@yahoo.com; 7 Department of Animal Production, Faculty of Agriculture, Tanta University, Tanta 31527, Egypt; 8 Department of Animal Production, Faculty of Agriculture, Kafrelsheikh University, Kafrelsheikh 33516, Egypt; 9 The Center for Applied Research on the Environment and Sustainability, The American University in Cairo, Cairo 11835, Egypt

**Keywords:** plant protein meal, red seabream, fish meal, health status, growth-promoting genes

## Abstract

**Simple Summary:**

Fish meal (FM) is the major protein source in aquafeed to achieve sustainable aquaculture production. However, the supply of FM is low due to high cost and low availability. There is ongoing conceited research to identify alternative viable protein sources to replace the finite FM. Novel camelina meal (CM) is a plant protein with high amino acids levels and has been tested as an alternative protein source in livestock feeding. However, there is limited information on supplementation of the CM in aquaculture diets. In this study, four diets were formulated to contain 0% plant protein, 205 g/kg soybean meal, and two levels of CM (205 g/kg and 330 g/kg) in diets fed to red seabream. Results indicated that adding CM 205 g/kg in diets-maintained growth performance, digestive enzyme activities, and nutrient digestibility regulated the immunity and stress resistance and modulated the growth-related genes in red seabream. These findings provide the first step in using novel CM and are essential for future practical formulations of feed for red seabream and other marine fish species.

**Abstract:**

A 60-day experiment was designed to assess the effect of different ratios of fish meal (FM): camelina meal plant protein (CM) on growth response and relative gene expression of growth-promoting factors, feed utilization potency, digestive enzymes activities, apparent digestibility (ADC), stress response, non-specific immunity of *Pagrus major*. Four isonitrogenous (490.7 g/kg of crude protein) and isolipidic (91.5 g/kg total lipid) experimental diets were formulated and designated as camelina meal (CM0), soyabean meal (SBM20.5), CM20.5, and CM33 based on protein contents. At the end of the feed trial, significantly higher (*p* < 0.05) weight gain, specific growth rate, and feed intake but lower feed conversion ratio were recorded in fish fed CM0, SBM20.5, and CM20.5 than fish fed CM33. The lowest growth, feed utilization, enzyme activity, and digestibility were recorded in fish fed CM33. Significantly higher pepsin, amylase, and protease activities were observed in fish fed CM0, SBM20.5, and CM20.5 diets than fish fed CM33. The highest ADC of protein was recorded in fish fed CM0, SBM20.5, and CM20.5 diets. Hematocrit levels were depressed CM33 while total serum protein, total cholesterol, triglyceride, blood urea nitrogen, total bilirubin, aspartate aminotransferase, and alanine aminotransferase were not significantly changed by the inclusion of CM. Non-specific immune variables (lysozyme activity, peroxidase activity in serum and nitro blue tetrazolium) in fish fed CM0, SBM20.5, and CM20.5 were significantly higher than in fish fed CM33 diet. The superoxide dismutase of fish fed CM20.5 was not significantly different from CM0 and SBM20.5 (*p* > 0.05). Catalase and low salinity stress test show that CM0, SBM20.5, and CM20.5 were not significantly (*p* > 0.05) different, while CM33 was significantly lower than the rest of the diets. TBARs show that CM20.5 and CM33 diets were significantly different (*p* < 0.05), but CM20.5 was not significantly different from SBM20.5. Significantly higher hepatic *IGF-1* and *IGF-2* mRNA expression was found in fish-fed diet groups CM0, SBM20.5, and CM20.5 than fish fed CM33. The present study indicated that the addition of CM up 205 kg/kg to diet maintains growth, digestive enzymes, nutrient digestibility, immunity, stress resistance, and feed utilization efficiency of red sea bream.

## 1. Introduction

Fish meal (FM) is a primary protein ingredient in aquaculture enterprises. Availability and accessibility of FM have been plummeting due to unprecedented demands for various uses in the wake of the global population boom [1]. Low FM supply has, in turn, caused prices to soar [2]. Food and Agricultural Organization (FAO) data released in 2016 projects that there will be a widening gap between accessible FM proportions and its usage in the ever-increasing global aquaculture dispensation [3]. Therefore, it is imperative to explore alternative protein sources in the aquafeed sector. For the past decade and a half, there has been a surge in research identifying alternatives to the finite FM. Numerous proteins have been examined and recommended for use in aquafeed to lessen the inclusion of FM while maintaining the growth performance and health of cultured animals [4]. *Hermetia illucens* prepupae meal in practical diets of rainbow trout (*Oncorhynchus mykiss*) [5], Housefly pupae (*Musca domestica*) [2], soy milk [6], fermented rapeseed meal [7], microalgae *Schizochytrium* species [8,9] are among the plant or other proteins that can partially replace FM in cultured red sea bream. Scientific evidence shows that considerable quantities of FM in diets of Atlantic salmon can be substituted with plant protein without showing deleterious growth response and nutrient use [10]. Conversely, other feed trials have presented the detrimental ramifications of using enormous proportions of plant protein in place of FM [11]. In addition, plant proteins have high proportions of anti-nutritional factors (ANFs) and fiber that diminish palatability, feed intake, and nutrient digestibility [12]. However, plant protein meals have a positive influence if incorporated in adequate quantities in animal diets. Notably, Sinigrin molecules present in plant meals have high antioxidative and immune efficacy that improve health status in foodstuffs [13]. Plant-based camelina meal (CM) is pegged at $260/metric ton compared to FM, which fetches $1385/metric ton [14] and therefore, the former is a viable and affordable alternative for the latter. Meanwhile, novel oilseed *Camelina sativa* has just been re-introduced in Canadian agriculture as an alternative source of biofuel, especially jet fuel [15]. Cruciferous (Brassicaceae) *Camelina sativa* thrives under low fertile and highly salty grounds; it is highly resilient to insect attack and does well in frost (freeze and thaw cycles) after they emerge late in cold and cool to warm climate [16]. Furthermore, the literature confirms the potential of camelina oil as a lipid source in aquaculture and livestock sectors [17,18,19]. A recent study by Mzengereza, et al. [20] show that complete substitution of fish oil by camelina oil in red seabream (*Pagrus major*) maintains growth performance and sustains health as far as limiting essential fatty acids, ecopentanoic acid (EPA), and docosahexanoic acids (DHA) are incorporated in diets of red sea bream. However, there is a handful of documented information on the efficacy of using the CM in aquaculture diets. Bullerwell, et al. [21] reported that there were desirable feed utilization efficiencies, intestinal morphology, proximate content of carcass, and growth performance in rainbow trout (*Oncorhynchus mykiss*) fed diets supplemented with up to 200 g/kg CM in its early trials and proposed further studies to determine optimum inclusion. Preliminary nutritional evaluation conducted by Hixson, et al. [22] show that CM has 38% crude protein and considerable proportions of methionine, lysine, phenylalanine, threonine, leucine, isoleucine, and valine. The residual crude lipid in the meal (16%) is rich in α-linoleic acid (18-3n-3) and linoleic acid (18 = 2n-6) fatty acids [23]. In addition, CM contains phytochemicals including phytic acid, total tannins, sinapines, and glucosinolates that interfere with animals’ chemical and physiological processes [24]. Alterations to the dietary composition of feedstuffs require prior knowledge of the nutrient utilization index of each ingredient, such as the apparent digestibility coefficients (ADCs) of the nutrients and digestive enzyme activities to avoid adverse repercussions on the overall performance of the cultured animals [25]. In invertebrates, stress response comprises many physiological processes regulated by the hypothalamus-pituitary-adrenal axis [26]. In fact, with teleost fish, it has been established that the inducement of both the hypothalamus and the pituitary controls cortisol release [26]. In particular, the adrenocorticotropic hormone (ACTH) is considered the most critical molecule that regulates the synthesis and the release of cortisol from the interrenal cells in the head kidney. Olivotto, et al. [26] reported that cortisol, glucocorticoid receptor, and *HSP70* are essential markers in fish acute stress response. Mzengereza et al. [20] reported that superoxide dismutase and catalase are the first line of defense enzymes that reflected the effects on the antioxidative state of red seabream after being fed diets incorporated with camelina oils.

Bio-molecular variables have gained credibility for aquaculture studies to supplement already available husbandry practices and evaluate farmed fish responses to various stimuli in their environment [27]. Evidence exists that biotechnology items (relative growth-related gene particularly insulin-like growth factor–I (*IGF-I*) and insulin-like growth factor-I (*IGF-II*) have been utilized as a bio-molecular tool to assess growth response in finfish experimentation [28] on the one hand and to examine the effectiveness of commercial diets in the short term on the other hand. In addition, *IGF-I* is an important metabolic pathway biomarker for molecular tissue in finfish growth; modifications in the *IGF-I* gene indicator can partially lead to alterations in growth performance [29]. Furthermore, *IGF-I*, mainly produced in the liver, is an essential parameter in the protein anabolism of fish [30]. The current feed trial was designed to determine the optimum supplementation level of CM for the red seabream diet and its ramifications on growth performance, expression of growth-promoting genes, feed utilization, body biochemical variables, stress tolerance capacity, and immune response.

## 2. Materials and Methods

### 2.1. Camelina Meal and Test Diets

Camelina meal (CM) was purchased from Tokai Seapro Co., Ltd. (Fukuoka, Japan) and finely ground into powder form using a blender. Four diets were formulated with uniform crude lipid and crude protein contents. Dietary treatments were graded as follows: control diet (fish meal, CM0) and experimental diets containing 205 g/kg soybean meal (SBM20.5), 205 g/kg CM (CM20.5), and 330 g/kg CM (CM33). The diets were formulated in line with the nutritional requirements of finfish enshrined in NRC [31] (Table 1). Dry ingredients were mixed for 45 min using an RM-G10 food mixer (Remacom Co., Ltd. Shizuoka, Japan) by slowly adding lipid sources initially premixed in a sonicator (USK-3RA, As One Corp., Osaka, Japan) and 30–40% water. The dough was extruded through a grinder with 1.2–2.2 mm in diameter to produce air-dried pellets in a DK 400 mechanical convection oven (Yamato Scientific, Tokyo, Japan) at 60 °C for 2 h. The diets with 10% moisture content were stored under −20 °C until and during the feeding trial. The feedstuffs, diets, and whole fish body were analyzed for moisture, crude protein, and ash using standard methods [32]. Total lipid (TL) contents of test diets and whole fish body were determined according to Bligh and Dyer [33]. The total amino acid (TAA) profile of the diet was determined using the liquid chromatography technique (HPLC, Shimadzu Corporation, Kyoto, Japan) following the procedure reported in Teshima et al. [34] and Kader, et al. [35] (Table 2). Firstly, 2 mg of sample was mixed with a known concentration of norleucine as internal standard, to this mixture, 4 N methanesulfonic acid was added and hydrolyzed for 22 h at 110 °C. Finally, the pH of the hydrolysate was adjusted to 2.2 ± 0.05, then filtered and stored at 4 °C. The separation and chromatography assessment of the amino acid content was conducted using an HPLC ion exchanging resin column.

### 2.2. Fish Husbandry and Sampling Methods

A feeding trial was performed on 120 red seabream *(Pagrus major*) (6.5 g average individual initial weight). The trial was done at the Kamoike marine production facility of the Kagoshima University Faculty of Fisheries (Kagoshima city, Japan). Fish were procured from a commercial fish hatchery in Miyazaki prefecture in Japan. Fish were acclimatized for four weeks to dissolved oxygen concentration between 6.6–6.1 mg/L and water temperatures range of 16.3–18.2 °C. Red seabream juveniles were unbiasedly stocked into twelve 100 L polyethylene circular tanks. Triplicate tanks per diet were stocked with 10 fish each in a flow-through system at natural photoperiod (12 h light/12 h darkness). Fish were fed twice daily to apparent satiation (8:00 am and 3:00 pm) per day for 60 days. After a 60-day feeding period, all fish were starved for 24 h before sampling was done. The fish were inoculated with eugenol (4-allylmethoxyphenol, Wako Pure Chemical Ind., Osaka, Japan) as an anesthetizer. All fish were weighed individually and counted for the evaluation of the growth performance and survival. Further, the whole-body length was detected for the calculation of the condition factor. Three fish per group (1 fish/tank) were collected and kept at −20 °C for proximate analysis using the standard method mentioned earlier. Nine fish per treatment (three fish/tank) were used for blood collection while their intestines, livers were dissected and kept for intestinal digestive enzymes, liver antioxidative capacity, and gene expression. The livers of fish were weighed to calculate the hepatosomatic index (HSI). Four fish per tank were used in the low water salinity stress (triplicates). The remaining fish per tank were collected and divided into two replicates per treatment to run the digestibility trial:

Weight gain (%) = (Weight _60 Days_ − Weight _0 Day_)/Weight _0 day_ × 100; (1)

Specific growth rate (SGR %/day) = ((Ln Weight _60 Days_ − Ln Weight _0 Day_)/60) × 100; (2)

Survival (%) = (Fish No. _60 Days_/Fish No. _0 Day_) × 100; (3)

Feed intake (g/fish/60 days) = (dry diet provided −dry uneaten diet retrieved)/no. of fish;(4)

Feed efficiency ratio (FER) = Fish live weight gain (g)/dry feed intake (g);(5)

(6) $$\mathrm{Condition}\;\mathrm{factor}\;\left(\mathrm{CF}\right)=\frac{W}{L^{3}}\times 100$$

(7) $$\mathrm{HSI}=\frac{\mathrm{Liver}\;\mathrm{weight},\;g}{\;\mathrm{Fish}\;\mathrm{body}\;\mathrm{weight},\;g\;}\times 100$$

### 2.3. Determination of Antinutrients Contents in Camelina Meal

Antinutrients were determined using procedures proposed in AOAC [32]. Briefly, trypsin inhibitors were assayed using casein solution added to all samples as substrate. Absorbance was read at 380 nm. Tannin standard solution (tannic acid) was prepared ranging (10–30 mg/L) to determine tannin. The absorbance of the standard solution and samples was monitored at 500 nm. Phytic acid was evaluated by 0.3% ammonium thiocyanate solution added into each sample as a marker and was titrated with iron (III) chloride solution (0.00l95 g iron/mL). The ultimate result is a somewhat brownish yellow color that lasts for 5 min. Protease inhibitors were measured using egg albumin as substrate (2% in phosphate buffer, pH 7), and 1 mL of the Bromelain enzyme solution (1% in phosphate buffer, pH 7) were incubated for 10 min at 55 °C. To stop the reaction, 5 mL of 10% trichloroacetic acid (TCA) was applied. The samples were read at 280 nm. All samples’ absorption was measured in the spectrophotometer (Spectronic 200, Thermo Fisher Scientific K.K., Tokyo, Japan). The results are displayed in Table 3.

### 2.4. Digestive Enzyme Assay

Fish were starved for 24 h before sample collection and collected intestines and stomach. The fish were inoculated with an anesthetizer (Eugenol, 4-allylmethoxyphenol, Wako Pure Chemical Ind., Osaka, Japan) before dissection. The sample was washed with 2% sucrose buffer and weighed storage at −81 °C until use. Intestine (wet wt. 2 g) were mixed with 5 mL of ice-chilled homogenization buffer (20 mM Tris-HCl, 1 mM EDTA, 10 mM CaCl_2_, pH 7.5) [32]. Samples were homogenized using a pellet pestle cordless motor (Sigma-Aldrich, St. Louis, MO, USA). 400 μL of Tris buffer was added and centrifuged for 30 min at 1700× *g*, 4 °C. The supernatant was preserved at −81 °C until enzymatic assay.

### 2.5. Protease Activity

Bradford’s method employing protein assay Coolmassie Brilliant Blue (CBB) solution (Nacalai Tesque, Inc., Kyoto, Japan) was done for protein content quantification. Protease activity was measured by Qing and Xing [36] method with some modifications using 0.5% casein as a substrate. The assay was performed as follows: 100 μL of 0.04 mol/L EDTA-Na, 2 mL of 0.5% casein, 400 µL of Tris buffer, 200 µL of enzyme extract sample of the intestine, and 800 µL distilled water were mixed and incubated for 15 min. The reaction was quenched by adding 1 mL of 30% cold TCA. The mixture was centrifuged at 2000× *g* for 20 min at 4 °C. One mL of the supernatant was mixed with 1 mL of Folin reagent and 5 mL of 0.55 mol/L Na_2_CO_3_, gently mixed and incubated for 15 min. OD was measured using a microplate reader (Multiskan GO, Thermo Fisher Scientific K. K., Tokyo, Japan) at 680 nm using distilled water as blanks. One unit was measured as the hydrolysis of casein that liberated 1 μg of tyrosine per min.

#### 2.5.1. Pepsin

Pepsin activity was performed according to Natalia et al. [37] using 2% hemoglobin in 0.06 NH_4_Cl as substrate. Five hundred μL of 2% haemoglobin in 0.06 N HCl substrate was mixed with 100 μL of crude enzyme extract of the stomach and incubated for 10 minutes at ambient temperature. The reaction was quenched by the inclusion of 1 mL of 5% TCA, and the mixture was incubated for 5 min at ambient temperature. The mixture was centrifuged for 5 min at 12,000× *g* under 4 °C. Optical density (OD) was recorded at 280 nm using a microplate reader (Multiskan GO, Thermo Fisher Scientific K. K.). For blank reading, trichloroacetic acid was used instead of enzyme extract.

#### 2.5.2. Lipase

Lipase activity was evaluated following the procedure of Roberts [38]. 4-methylumbelliferyl butyrate (4 MUB) substrate was applied. 60 μL of the substrate (0.5 mM 4 MUB, 5 mM egg lecithin, 10 mM sodium taurocholate, and 150 mM NaCl) was mixed with 20 μL of Tris buffer (pH 7.5) and 20 μL of crude enzyme extract. Each sample was divided into two, each placed at 4 °C in an ice bath and 37 °C in a water bath and simultaneously incubated for 10 minutes. 0.2 mL Tris buffer (1 M, pH 7.5) was added to stop the reaction. Emission was done at 450 nm (excitation 380 nm) using a fluorescence spectrophotometer (F-2700, Hitachi High-Tech Corp. Tokyo, Japan). The difference in fluorescence between the readings taken on the 4 °C incubated samples and the 37 °C control samples per assay were the final reading. Basal stock solutions of 4-methylumbelliferone were used as blank.

#### 2.5.3. Amylase

Amylase potency was determined using the modified method proposed by Murashita, et al. [39], where 1% starch solution was used as a substrate. Fifty microliters of enzyme extract, 25 μL 20 mM sodium phosphate buffer (pH 6.9, containing 6.0 mM NaCl), and 25 μL of the substrate solution were mixed and incubated at 37 °C for 60 minutes. Fifty μL of dinitrosalicylic acid reagent (1% dinitrosalicylic acid and 30% sodium potassium tartrate in 0.4 M NaOH) was added to the mixture to quench the reaction and samples were incubated in boiling water for 5 min. The OD was measured using a microplate reader (Multiskan GO, Thermo Fisher Scientific K. K.) at 540 nm using a maltose solution as blank. Total liberated maltose was evaluated by the standard curve. The activity was measured in units of U, which equaled the quantity of maltose released in one minute (mol).

### 2.6. Blood Function Assessment

After the growth experiment, fish were sampled by drawing blood from the caudal vein. Heparinized disposable syringes were used for collecting blood for hematocrit and other plasma bioassays, while non-heparinized disposable syringes were used to collect blood for serum analysis. Plasma and serum samples were centrifuged at 3000× *g* for 15 min at 4 °C using a centrifuge (MX-160; Tomy Seiko Co., Ltd., Tokyo, Japan) and kept at −80 °C for later use. Hematocrit was assayed in the microhematocrit machine using whole blood. Serum parameters were assessed by a dry chemistry analyzer (SPOTCHEMTM EZ model SP-4430, Arkray, Inc. Kyoto, Japan). 

### 2.7. Non-Specific Immunological and Antioxidative Assays

Serum total peroxidase was determined by referring to a procedure by Salinas et al. [40]. Lysozyme activity in serum was determined turbidimetrically as stipulated in protocols of Lygren, et al. [41]. An enzyme activity unit was defined as the amount of enzyme that produced a decrease in absorbance of 0.001/min. The oxidative radical production by neutrophils during respiratory burst was measured by the nitro blue tetrazolium (NBT assay in whole blood samples [42]. Biological antioxidant potential (BAP) and reactive oxygen metabolites (d-ROMs) were also measured spectrophotometrically in blood plasma with an automated analyzer (FRAS4, Diacron International s.r.l., Grosseto, Italy) following procedures recommended by the commercial FRAS4 manufacturer, d-ROMs test, and BAP Test, (Wismerll Co., Ltd., Tokyo, Japan). Antioxidant enzyme function was measured in liver tissues after the experiment. Fish were killed in a slurry of ice water, dissected, and liver collected and kept at −80 °C until used for analysis. Liver samples were firstly homogenized in sucrose buffer and centrifuged at 4 °C and 12,000 rpm for 10 min. Superoxide dismutase (SOD) activity was determined using the SOD-WST assay kit (Dojindo Molecular Technologies, Inc. Kumamoto, Japan) and absorbance read at 450 nm. The catalase activity (CAT) assay was performed using spectrophotometric determination of hydrogen peroxide (H_2_O_2_), which forms a stable complex with ammonium molybdate that absorbs at 405 nm. The thiobarbituric acid reactive substances (TBARs) concentration was measured using a TBARs assay kit (Cayman Chemical, Miami, FL, USA) at 540 nm. The supernatant of liver samples was determined using a microplate reader (Multiskan GO; Thermo Fisher Scientific, K.K.).

### 2.8. Low Salinity Stress Evaluation 

Four fish per tank were placed in 20 L transparent glass tanks at random. The 20 L tanks contained 18 L of dechlorinated freshwater. This test was conducted in triplicate for each experimental treatment. Time taken to reach 50% mortality was calculated by the following equation [43]:

Y = aX + b(8)

where Y = log10 (survival), X = time to individual death of fish (min). LT_50_ (X) was obtained when Y = 1.7 as log_10_ (50) = 1.7. 

### 2.9. Digestibility Assessment

The digestibility of nutrients (crude protein, crude lipid, and dry matter) was measured by an indirect method using chromium oxide as an inert marker. Before feces collection, juvenile red seabream was accustomed to the diet containing chromic oxide for six days. Feeding schedules to apparent satiation twice daily were maintained. Faecal matter siphoned from the bottom and collecting them into the small fishnet. Faeces were pooled and stored at −20 °C until analysis. Soon before analysis, faeces were freeze-dried in and milled to powder form. Levels of chromium oxide in diets and faeces were quantified by Furukawa [44]. The following formulas were used to computerize the ADCs of the nutrients.

ADC_nutrient_ (%) = 100 − (% Cr_2_O_3_ diet/%Cr_2_O_3_ faeces ×% nutrient faeces/% nutrient diet) (9)

ADC_drymatter_ (%) = 100 − (100 − (% Cr_2_O_3_ diet/% Cr_2_O_3_ faeces) (10)

### 2.10. Real Time PCR Analysis

Fish were dissected, and liver samples were obtained. Liver samples were placed in fivefold of RNAlater (Invitrogen; Thermo Fisher Scientific K. K.) liver weight and stored at −80 °C until analysis. The RNeasy Mini Kit 50 (Qiagen; Hilden, Germany) was used for the RNA extraction. Briefly, liver samples (30 mg) were placed in a tube, homogenized, and centrifuged at 12,000 rpm for 15 s. The supernatant was collected and mixed with 70% ethanol. After RNA extraction, cDNA was obtained using the Prime ScriptTM RT Master Mix Kit (Takara Bio Inc. Shiga, Japan), following the manufacturer’s protocol. Real-time PCR analysis was performed using SYBR Select Master Mix kit (Thermo Fisher Scientific K. K.) using the following primers as presented in Table 4. 

Elongation factor (β-actin) was used as the housekeeping gene (Table 4). Amplification was performed using the CFD-3120 Mini Opticon Real-Time PCR System (BIO-RAD, Singapore) with the following protocol: Initial 2 min denaturation at 95 °C, 40 cycles of 95 °C for 15 s, and 65 °C for 30 s. Each assay was done in triplicate 0 °C for 30 s.

### 2.11. Statistical Analysis

Firstly, the normal distribution of the data was verified using Kolmogorov–Smirnov test and the Shapiro-Wilk test. To confirm the homogeneity of variance on data sets, it was subjected to the Levene test. The experimental tanks were arranged in a Completely Randomized Design (CRD) and treatment diets replicated; therefore, the experimental design fulfilled the third assumption of using ANOVA, which demands that observations be independent of each other. Data were then subjected to a one-way analysis of variance (ANOVA). All analysis was performed using Super ANOVA 1.11 (Abacus Concepts, Berkeley, CA, USA). Probabilities of *p* < 0.05 were defined as significant. Significant different means among various treatments were evaluated using the Tukey– Kramer post hoc test. Results are reported as the “mean ± S.E.” (S.E referring to the standard error of the mean). To synthesis the data, multivariate analyses were performed on the data to summarize the overall response of red seabream to the test diets. Briefly, data was holistically normalized and plotted using heat map graphical function readily developed in GraphPad Prism version 8.0.1 for Windows (Graph Pad Software, San Diego, CA, USA). To run by principal component analysis (PCA), data was standardized followed by PCA. Data were finally visualized by running the Unweighted Pair Group Method with Arithmetic Mean (UPGMA) hierarchical algorithm using a correlation matrix model. PCA and UPGMA were analyzed using Paleontological Statistical Software version 3.21, University of Kansas, Lawrence, KS, USA [46].

## 3. Results

### 3.1. Growth and Nutrient Utilization Variables

Information on growth response, feed efficacy indices, and survival of fish were shown in Table 5. 

By the end of the 60-day feeding period, fish served control, SBM20.5, and CM20.5 diets showed significantly higher final live body weight, weight gain, specific growth rate, and feed intake, but lower feed conversion ratio than fish fed CM33 diet. Survival rates, hepatosomatic index, and condition factor rates of fish were not markedly changed by the supplementation of CM in the diets.

### 3.2. Whole Body Proximate Evaluation

Carcass proximate composition of *Pagrus major* juvenile is presented in Table 6. There was no alteration (*p* > 0.05) on ash and lipid contents at the end of 60 days of the feeding regime. The group fed CM33 showed lower whole-body protein levels than all diets, including control (*p* < 0.05).

### 3.3. Digestive Enzyme Activity

Table 7 represented the enzyme activity in the intestine of red seabream fed test diets. Intestinal enzyme activities (protease, lipase, and amylase) and stomach enzyme activity (Pepsin) in fish fed CM33 recorded the lowest values compared to the other group. Except for lipase, CM has an impact on enzyme activities in the intestine of red sea bream.

### 3.4. Serum Biochemical Constituents

Table 8 carries the serum biochemistry data of red seabream juveniles after the 60-day feeding period. Hematocrit levels were depressed CM33 (*p* < 0.05) when compared with the control group. Except for the glucose, total serum protein (T-Pro), total cholesterol (T-Cho), triglyceride (TG), blood urea nitrogen (BUN), total bilirubin (T-Bill), and aspartate aminotransferase test (AST) and alanine aminotransferase test (ALT) were not significantly (*p* < 0.05) changed by the inclusion of CM. 

### 3.5. Immunological Response 

**Figure 1:**
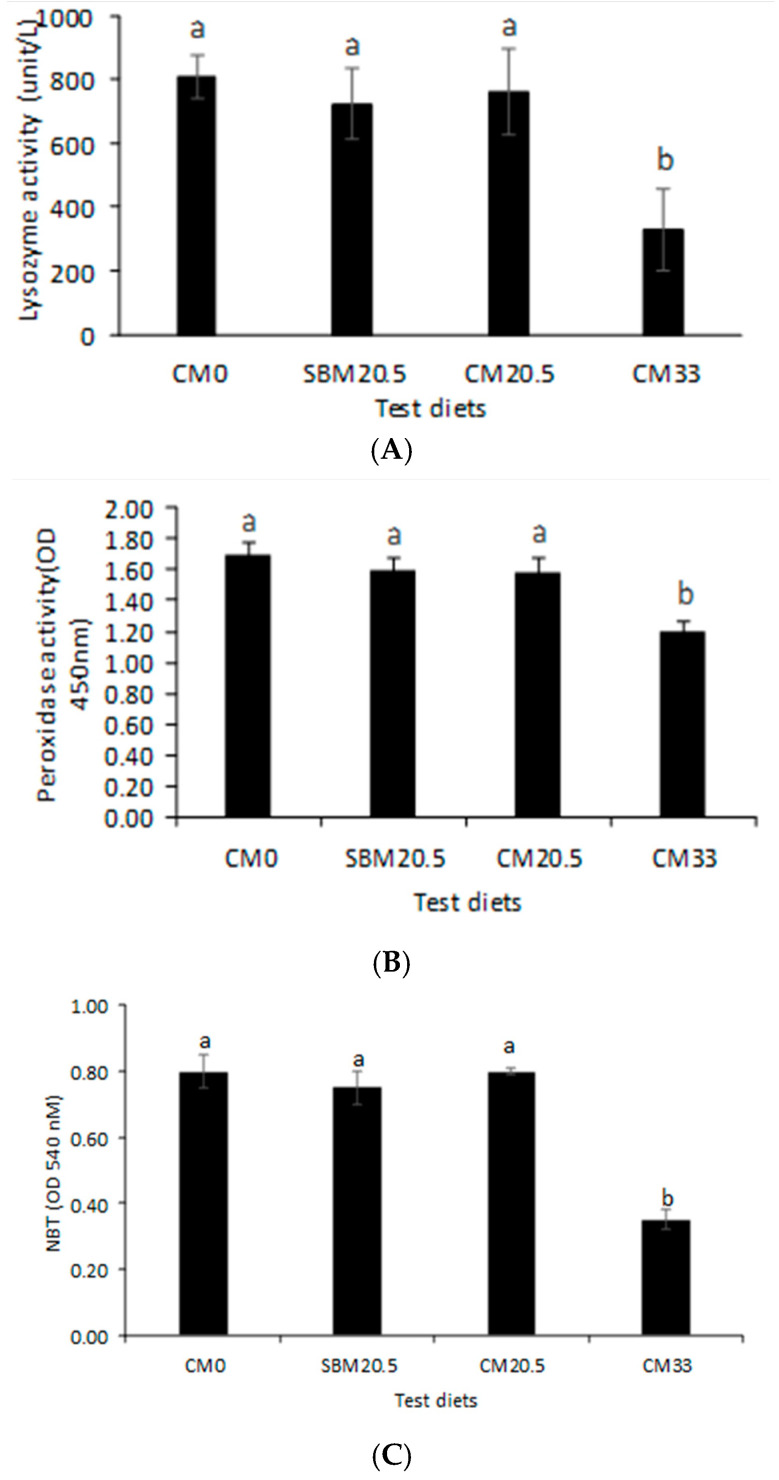
(**A**) Serum lysozyme activity (U/L, *n* = 3); (**B**); serum peroxidase activity (*n* = 3); (**C**) nitro blue tetrazolium (%, mean ± standard error, *n* = 3) of nutrients in red seabream fed diets formulated by replacing fish meal with camelina meal at 0% for reference diet (CM0), 20.5 g/kg with soybean mean (SBM20.5), 20.5 g/kg with camelina meal (CM20.5) and 33 g/kg camelina meal (CM33).Values with different letters are significantly different (*p* < 0.05).

Figure 1 presents the non-specific immune response data of red seabream juveniles after the 60-day feeding on CM-rich diets. 

Results show that lysozyme activity, peroxidase activity in serum, and nitro blue in whole blood of red seabream fed up to 205 g/kg CM were not significantly different from control (CM0) and soybean meal diet (SBM20.5) (*p* < 0.05). In contrast, fish serum-fed meal camelina supplemented at 330 g/kg (CM33) were significantly lower (*p* > 0.05) than fish groups from the rest of the diets.

### 3.6. Resistance to Oxidative and Salinity Stress

**Figure 2:**
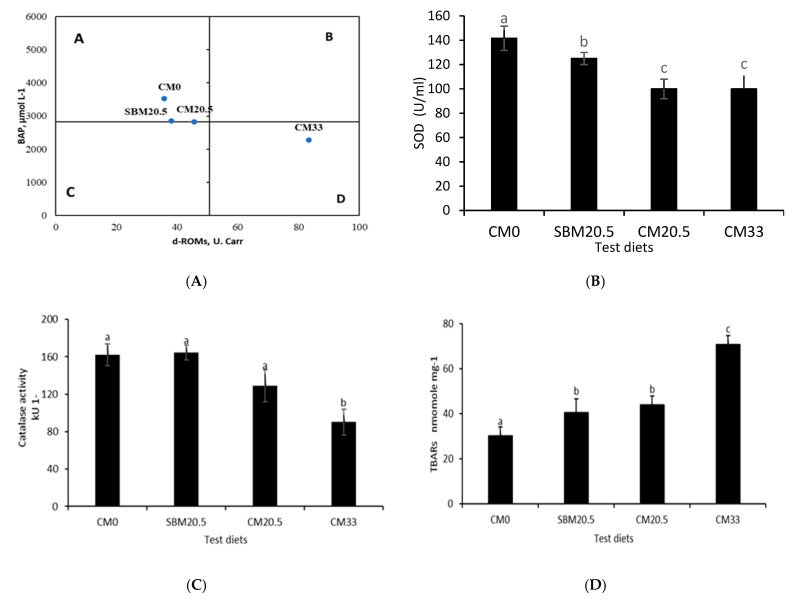
(**A**) BAP: Biological Antioxidant potential and d-ROM s; Reactive Oxygen Metabolites, (**B**) SOD; superoxide dismutase, (**C**) CAT, Catalase (**D**) TBARs: Thiobarbituric Acid Reactive substances (%, mean ± standard error, *n* = 3) of nutrients in red seabream fed diets formulated by replacing fish meal with camelina meal at 0% for reference diet (CM0), 20.5 g/kg with soybean mean (SBM20.5), 20.5 g/kg with camelina meal (CM20.5) and 33 g/kg camelina meal (CM33). Values with different letters are significantly different (*p* < 0.05).

**Figure 3:**
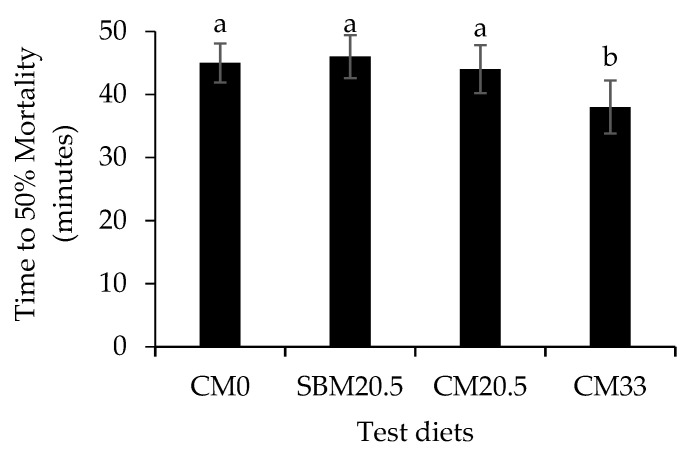
LT_50_ (min) was obtained from the onset of mortality time of red sea bream exposed to freshwater. (%, mean ± standard error, *n* = 3) of nutrients in red seabream fed diets formulated by replacing fish meal with camelina meal at 0% for reference diet (CM0), 20.5 g/kg with soybean mean (SBM20.5), 20.5 g/kg with camelina meal (CM20.5), and 33 g/kg camelina meal (CM33). Values with different letters are significantly different (*p* < 0.05). Values with different letters are significantly different (*p* < 0.05).

Figure 2 illustrates antioxidant enzymes function and thiobarbituric acid reactive substances (TBARs) data, while Figure 3 shows low salinity challenge results of red seabream juveniles after the 60-day feeding on camelina meal rich diets. Values are presented in triplicates = 3. Central axis based on mean values of d-reactive oxygen metabolites (d-ROMs) and biological antioxidant potential (BAP) from each treatment. Results show that in zone A, high (BAP) and low d-ROMs (good condition) for fish groups CM0, SBM20.5, and CM20.5 and Zone D low BAP and high d-ROMs (stressful condition) for CM33. Results for superoxide dismutase (SOD) and show that red seabream fed up to 205 g/kg CM were not significantly different from control (CM0) and (SBM20.5), but CM20.5 and CM33 were not significant (*p* > 0.05). Catalase (CAT) and low salinity stress test show that CM0, SBM20.5, and CM20.5 were not significantly (*p* < 0.05) different from each other, while CM33 was significantly lower than the rest of the diets (*p* > 0.05). TBARs show that camelina diets CM0, CM20.5, CM33 were all significantly different (*p* <0.05), but CM20.5 was not significantly different from SBM20.5.

### 3.7. Apparent Digestibility Coefficients of Nutrients 

The findings of apparent nutrient digestibility showed that there was no significant (*p* > 0.05) differences in total lipid and dry matter among the fish group (Table 9). In CM0, SBM20.5, and CM20.5, similar protein digestibility was observed. However, protein digestibility in CM33 was significantly (*p* < 0.05) lower among the other diets. 

### 3.8. Relative Growth Gene Expression

**Figure 4:**
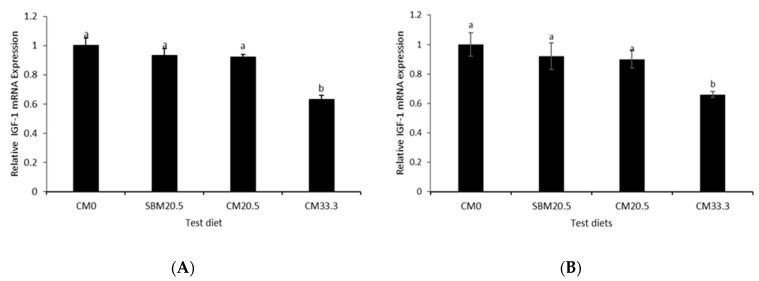
(**A**,**B**) qPCR analyses of relative expression of growth-related genes (*IGF-1* and *IGF-2*) in the liver of red seabream fed four different diets by replacing fish meal with camelina meal at 0% for reference diet (CM0), 20.5 g/kg with soybean mean (SBM20.5), 20.5 g/kg with camelina meal (CM20.5) and 33 g/kg camelina meal (CM33). Relative mRNA expressions results are presented in triplicated, *n* = 3 for all treatments. Letters indicate Turkey-Kramer post hoc test and confidence interval of 95% (*p* > 0.05) interpreted as Values represent means of triplicate groups ± S.E.M., *n* = 3 means with different letters are significantly different (*p* < 0.05); means with the same letters are not significantly different (*p* > 0.05). S.E.M. represents the standard error of mean.

Relative gene expression of hepatic *IGF-1* and *IGF-2* were presented in Figure 4A,B. Significantly higher hepatic *IGF-1* and *IGF-2* mRNA expression was found in fish-fed diet groups CM0, SBM20.5, and CM20.5 than fish fed CM33.

### 3.9. The Heatmap Analysis

**Figure 5:**
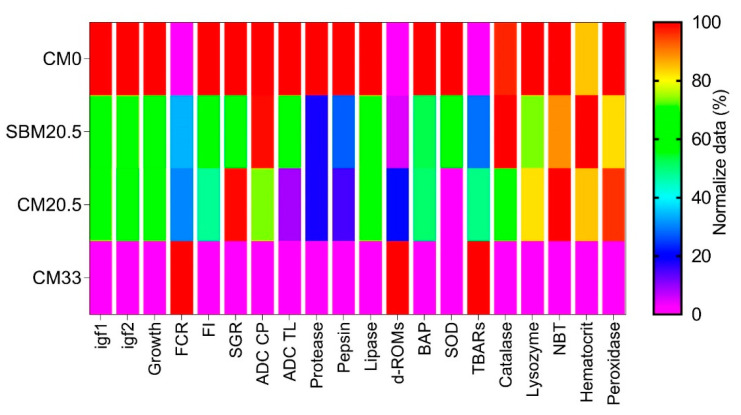
Heatmap diagram of red seabream fed four different diets by replacing fish meal with camelina meal at 0% for reference diet (CM0), 20.5 g/kg with soybean mean (SBM20.5), 20.5 g/kg with camelina meal (CM20.5), and 33 g/kg camelina meal (CM33). The horizontal axis showed the parameters being assessed, and the vertical axis showed the dietary groupings. The legend showed the normalized (0–100% scale) mean values of each parameter in the investigation. The red bar and purple bar represent the highest and lowest mean responses, respectively. In the Figure 5 abbreviations in the horizontal axis are define as follows: igf1 and igf2 represent growth factor gene 1 and growth factor gene 2 respectively, Growth: Final weight growth weight, FCR: Feed Conversion ratio FI: Feed intake, SGR: specific growth rate, ADC CP: Apparent nutrient digestibility of protein, ADC TL: Apparent nutrient digestibility of Total Lipid, d -ROMs: Reactive Oxygen Metabolites, BAP: Biological Antioxidant Potential, SOD, Superoxide Dismutase, TBARs, Thiobarbutic Reactive Substances, NBT: Nitro blue Tetrazolium.

In the Head map diagram (Figure 5), there was a marked distribution of response to different parameters in red seabream among four treatments. Comparatively, red seabream fed diet 0 g/kg camelina meal (CM0) without plant protein supplementation responded highly (100–80% mean distribution) in terms of overall growth parameters, oxidative resistance and freshwater stress responses, immune makers, feed utilization, and gene expressions than in red seabream compared with the diets supplemented with soybean meal and camelina meal. 

There is a comparable trend in growth performance, immunity, stress resistance, and feed utilization efficacy in red seabream fed diets with 205 g/kg Soybean or 205 g/kg camelina meal (30–80% mean distribution). As shown, supplementation with 330 g/kg camelina meal (CM33) reduced (less than 30% mean distribution the growth performance, digestibility, enzymes efficacy, nutrient utilization index, immunity, and stress capacity (Figure 5). 

### 3.10. Principal Components Analysis (PCA)

**Figure 6:**
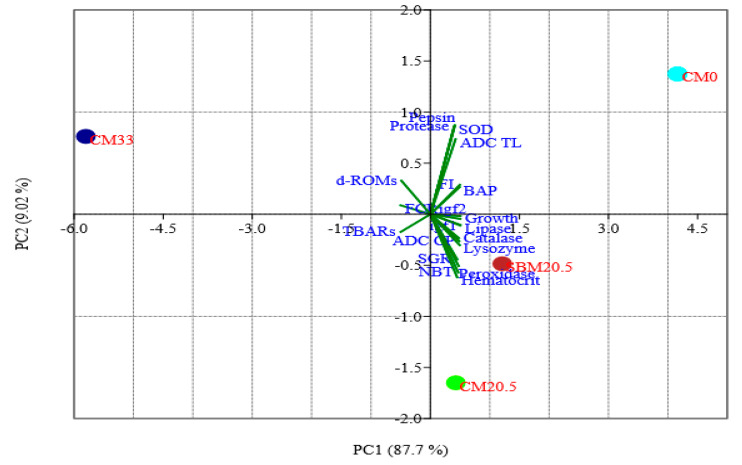
Principal component analysis (PCA) plot (PC: 1:87.7%, PC: 9.04%) correlation of responses of growth, feed utilization indices, immunity markers, stress biomarkers, relative gene expressions, digestibility coefficients, and enzyme activity of red seabream using a covariance, where fours diet treatments formulated by replacing fish meal with camelina meal at 0% for reference diet (CM0), 20.5 g/kg with soybean mean (SBM20.5), 20.5 g/kg with camelina meal (CM20.5) and 33 g/kg camelina meal (CM33).

The principal component analysis (PCA) presented in Figure 6 revealed that PC1 and PC2 components explained 97% (87.7% and 9.04%, respectively) of the total variation. The use of PCA in this study helps to explore existing correlations in relationships between parameters assessed for fish fed different diets. The loading plot helps to show the contribution of each variable to the difference between diets (Table 10).

The most significant positive correlations coefficients with the PC1 axis were found in the following parameters: feed intake (FI) growth response (IGF-1, IGF-2, SGR, growth (weight gain)), digestive enzymes (protease, pepsin, lipase), immune response (lysozyme activity, NBT, peroxidase, haematocrit) and antioxidant (catalase, BAP and SOD), apparent digestibility coefficients (ADC total lipid, ADC crude protein). The parameters that correlated most strongly negatively correlated with the PC1 axis were antioxidant (TBARs and d-ROMs) and FCR. The strongest positive correlations with the PC2 axis were found in the parameters: SOD, protease, pepsin, and ADC (TL). The parameter most strongly negatively correlated with the PC2 were SGR, NBT, haematocrit, and peroxidase. These parameters assessed in this investigation were associated with diets (CM0, SBM20.5, CM20.5, and CM0) and feed intake (Figure 6). Antioxidant (BAP, SOD), digestive enzyme (protease, pepsin), digestibility (TL), and growth (BW, IGF-2) enzyme were associated with CM0. Immune (lysozyme, NBT, catalase, peroxidase), blood health (haematocrit), and digestibility (CP) were associated with diet SBM20.5 and CM20.5. The FCR, d-ROMs, and TBARs were associated with CM33 (Figure 6). 

### 3.11. UPMGA Correlation Matrix

**Figure 7:**
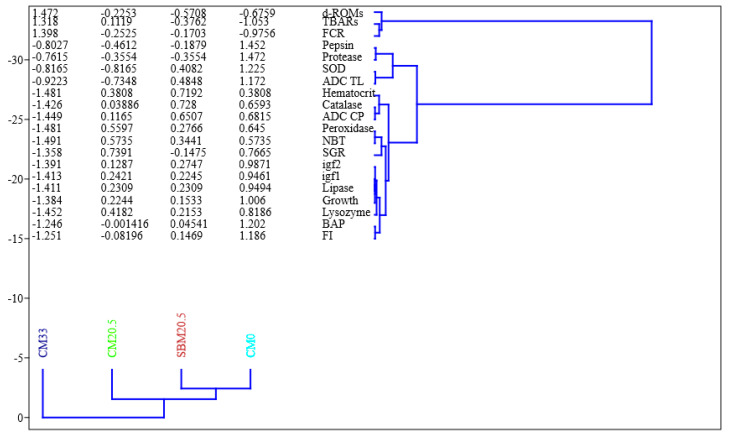
Dendrogram derived from UPMGA analysis of responses of growth, feed utilization indices, immunity markers, stress biomarkers, relative gene expressions, digestibility coefficients, and enzyme activity of red seabream using a correlation matrix model, where fours diet treatments formulated by replacing fish meal with camelina meal at 0% for reference diet (CM0), 20.5 g/kg with soybean mean (SBM20.5), 20.5 g/kg with camelina meal (CM20.5) and 33 g/kg camelina meal (CM33).

According to Figure 7, the following parameters: pepsin, protease, SOD, FCR, FI, *IGF-1*, *IGF-2*, d-RMOs, growth (weight gain), SGR, ADC (TL), Lipase, BAP, catalase, peroxidase, NBT, SGR had a strong correlation on CM0. Haematocrit, d-ROMs, catalase, ADC (CP) were strongly correlated to SBM20.5. Peroxidase, NBT, SGR are correlated to CM20.5. d-ROMs, FCR, and TBARs, correlated with CM33. The higher the correlation coefficient, the more the influence on the diet.

## 4. Discussion

Feed is a significant contributor to high aquaculture production costs. As a result, there is a global effort to identify affordable inputs to partially substitute expensive aquaculture and animal production feed sources, mainly fish meal [47]. Plant products have recently emerged as potential sources to replace fish meal in aquafeed [12]. The higher inclusion of camelina meals in diets of red seabream decreased growth response, weight gain (WG), and feed intake (FI) while increased feed conversion ratio (FCR) in the present experiment. Inadequate fish growth response observed at CM33 supplementation corroborates earlier work on the supplementation of plant protein in fish feed experimentation [48]. However, FM replacement with CM up to 20.5 g/kg positively affected fish final body weight, WG, specific growth rate, or FI. Bullerwell, et al. [21] recommended that the maximum dietary inclusion threshold of camelina meal in juvenile rainbow trout diets is 200 g/kg, although further research will be required to determine more definite values. Pan, et al. [49] found that dietary inclusion levels of high-oil residual camelina meal (HORM) up to 160 g/kg did not negatively affect the growth performance of rainbow trout. 

In the present study, despite the experimental diet meeting the amino acid requirement of red seabream, poor growth performance, FI, and FCR were still observed in CM33. In addition, enzyme activities in the intestine and digestibility of nutrients were lowest among the group. We believe that the dismal growth performance and poor feed utilization obtained in CM33 were caused by high amounts of phytochemicals, which are conventionally referred to as anti-nutritional factors (ANFs). While there are significant nutritional profiles in terms of protein and lipid levels in camelina meal, limitations for its utilization in aquafeed is the presence of ANFs that diminishes feed palatability and digestion of the feed [50]. Previous reports showed that ANFs in camelina include glucosinolates, phytic acid, sinapine, and tannins [50]. Bullerwell, et al. [21] reported that a significantly reduced feed intake of rainbow trout fed 300 g/kg camelina may have been due to high glucosinolate levels of this diet. Condition factor and viscerasomatic index (VSI) were not affected by dietary CM in red seabream. This result corroborates previous work on plant protein fed to red seabream [25]. For utilization of CM on aquaculture feeds, improvement of palatability using attractants and reduction of ANFs in CM by an extruder treatment or fermentation are necessary.

Results of the current study reveal that protein carcass proximate composition was significantly lowered by dietary CM. Thus, carcass compositions were similar to results that have been reported previously in red seabream [7]. Significant influences of dietary rapeseed plant protein lowered crude protein, crude lipid, and energy levels whole body in a study by Kader, et al. [35], similar to the present study. Therefore, we suggest that higher supplementation of CM at CM33 was a critical factor that lowered protein levels and subsequently resulted in poor growth of red sea bream. In contrast, our results agree with Bullerwell, et al. [21] and Pan, et al. [49], who observed that camelina meal inclusion at 200 g/kg and 160 g/kg respectively had a similar carcass composition as control-fed fish.

Endogenous enzymes are fundamental in digesting nutrients in experimental finfish to increase the bioavailability of micronutrients for the sustainability of physiological body needs [51]. Findings of the current feed trial have confirmed that higher proportions of CM (CM33) led to the lowest activity of all digestive enzymes analyzed except lipase activity. Deterioration of enzyme activity was inferred from ANFs, enormous proportions of fiber, and secondary plant metabolites in CM, diminishing nutrient digestion [52,53]. Remarkably, the dismal levels of ADC of crude protein in red seabream fed diets with high dietary (CM33) levels may have caused a reduction in pepsin and protease activities caused by protease and trypsin inhibitors present in CM.

The composition of feed ingredients has substantial ramifications on the ADC of various nutrients, notably crude protein, total or crude protein, energy, and dry matter [25]. In the present study, diets CM0, SBM20.5, and CM20.5 had significantly superior protein digestibility than diet CM33.3. Similarly, total lipid ADCs were numerically higher for diets CM0, SBM20.5, and CM20.5. No variance was detected in dry matter ADCs in fish fed different camelina diets. These results show that red seabream could not efficiently use increased proportions of CM, leading to growth retardation. The finding in the current feed trial agrees with Nagel, et al. [54], who reported that rapeseed incorporated diets resulted in low feed ingestion and poor nutritional quality of the diets for juvenile turbot. According to Kokou and Fountoulaki [55], plant-based protein sources contain proportions of anti-nutritional forms that lower nutrient digestibility. 

Principal component analysis (PCA) (Table 9, Figure 6), Heatmap (Figure 5), UPMGA Correlation matrix (Figure 7) data presented in this manuscript indicate that relative expression of growth-related genes; *IGF-1* and *IGF-2* were higher in CM0, SBM20.5, and CM20.5 and significantly lower on CM33 diets. This result is consistent with earlier work on the efficacy of liver *IGF-1* and *IGF-2*, which showed that relative expression of *IGF-2* was most significant in the liver of rainbow trout fed the control FM diet and lower in soybean diets, thus asserting that elevated *IGF-2* resulting in higher somatic growth in fish [56,57]. Similarly, there was a sharp downregulation of liver *IGF-I* gene expression in a fish-fed diet with elevated plant protein levels [58,59]. Therefore, the present study hypothesizes that there was marked depression of protein synthesis with marginal addition of CM in diets, which resulted in growth retardation of red sea bream. The expression of GH/IGF genes is amplified under optimum nutrients, frequent daily feeding [60], higher lipid-protein nutrients ratio [61], or the higher inclusions of dietary fishmeal [62].

Blood chemistry variables are regarded as the first indicator for the physiology, bio-chemical and pathological status of cultured animals in response to different feeding regimes [45]. Hematocrit and plasma glucose levels in CM33 showed significantly lower and higher respectively among the groups. Plasma glucose is generally considered an indication of stress in fish; low glucose levels indicate the low-stress status of fish [63]. Significantly lower glucose contents in fish-fed diets CM0, SBM20.5, and CM20.5 indicated that red seabream fed these diets displayed better physiological conditions than the group fed CM33.3. Lower hematocrit levels in CM33 support the previous results that fish groups were in poor health, and in contrast, results show that CM0, SBM20.5, and CM20.5 inclusion of CM in diets of red seabream registered higher hematocrit score thus maintained a normal physiological status.

SBM20.5 and CM20.5 diets groups maintained non-specific immune response, lysozyme activity, nitro blue tetrazolium reduction test (NBT), and peroxidase activity. Conversely, diet CM33 registered significantly lower non-specific immune indicators. Lin and Luo [64] highlight that high substitution proportions of FM by soybean meal diminished the immune system of tilapia by lowering lysozyme activity in serum. We speculate, therefore, that CM33 leads to the declination of growth performance, among other reasons, due to the poor health of fish.

Our findings show that as dietary CM levels increased, there was a corresponding decrease in liver SOD and CAT activities, while the amount of TBARs in the liver significantly increased. The results show that TBARs content was significantly higher in fish fed the SBM20.5, CM20.5, and CM33 diets than the control diet (CM0). The implication of our findings hypothesizes that high dietary CM and SBM levels may reduce the antioxidant capacity of juvenile red sea bream, thereby breaking the fluctuating cycle of synthesis and deamination of free radicals in organism hepatic oxidative stress induced by the inclusion of high levels of CM. Shen, et al. [65] found that the substitution of cottonseed protein concentrates for a proper amount of fish meal increased SOD activity. The previous antioxidative responses are supported by BAP and d-ROMs results, showing that diets CM0, SBM20.5, and CM20.5 showed high BAP and low d-ROM while CM33 showed low BAP and high d–ROM. The lower antioxidant response in CM33 groups could be the detrimental effects of the increased level of antinutrient levels products present in CM at high supplementation.

In fish farming, growth and stress response management is of primary importance since they affect production and farm income [66]. Stress indicates hormonal status, nutrient metabolism rate, feed utilization capacity, and overall immunity of cultured fish [66]. Acute and lethal stress challenge is used to examine general welfare and health status by measuring the lethal time of 50% mortality (LT_50_) in the freshwater of the fish [67]. Stress is a precursor for high energy demand in response to stimuli at the expense of anabolic processes, affecting survival and growth performance [68]. In the present trial, significantly higher values of LT_50_ obtained in diets CM0, SBM20.5, and CM20.5 indicated high resistance to salinity’s acute stress in fish groups fed these diets. On the contrary, fish fed diet CM33 produced lower values of LT_50_, indicating that a high level of CM supplementation diminished the tolerance of freshwater stress in red seabream. The capacity of fish to repel and adapt in a stressful environment is directly related to enhancing immune capabilities that enable resistance to various stressors and improves growth [69]. Piccinetti et al. [66] reported that the low cortisol levels detected at the earliest larval stages reflected a stress-coping ability. This feature may protect larvae from the high metabolic demands involved in stress responses, thereby promoting faster growth and survival of Ballan wrasse (*Labrus bergylta*). 

## 5. Conclusions

In a nutshell, the findings of the current study showed that CM protein could replace up to 20.5 g/kg of FM protein according to growth-related gene expression, growth performance, non-specific immunity, and protein enzyme activity as well as protein ADCs. However, high levels of FM protein substituted by CM protein in diets could decrease the growth performance and hepatic *IGF-I* gene expression level of red sea bream. Results herein provide new insight toward the optimization of alternative plant protein for the proper growth and development of farm-raised fish for human consumption and underscore the ability of camelina protein sources to deliver balanced nutrition. Further studies on protein concentration and the effects of the ANF in these products may increase the maximum inclusion level of camelina protein products in red seabream and feeds.

## Figures and Tables

**Table 1:** The proportion of ingredients (g/kg) and chemical composition (%) of experimental diets.

Ingredient (g/kg DM)	CM0	SBM20.5	CM20.5	CM33
Brown fish meal ^a^	59.0	45.0	45.0	31.0
Soybean meal ^b^	-	20.5	-	-
Camelina meal ^c^	-	-	20.5	33.0
Casein ^d^	1.0	1.0	1.0	6.0
Squid meal ^e^	5.0	5.0	5.0	5.0
Krill meal ^f^	4.0	4.0	4.0	4.0
Soybean lecithin ^g^	3.0	3.0	3.0	3.0
Pollack liver oil ^h^	2.0	2.0	2.0	2.0
Wheat flour ^i^	1.4	1.4	1.4	1.4
Activated gluten ^j^	5.0	5.0	5.0	5.0
Mineral mix ^k^	3.0	3.0	3.0	3.0
Vitamin mix ^l^	3.0	3.0	3.0	3.0
Stay C ^m^	0.08	0.08	0.08	0.08
Alpha-cellulose ^n^	13.52	7.02	7.2	4.00
Methionine	0	0.1	0.2	0.5
Lysine	0	0.09	0.29	0.65
Taurine	0	0.17	0.18	0.29
Total	100	100	100	100
Proximate constituents				
Crude protein	49.6 ± 0.4	49.9 ± 0.2	48.2 ± 0.9	48.6 ± 0.1
Total lipid	9.5 ± 0.1	9.4 ± 0.0	9.0 ± 0.0	8.7 ± 0.0
Moisture	11.2 ± 1.0	10.86 ± 0.3	10.95 ± 0.6	11.26 ± 0.5
Ash	12.4 ± 0.3	12.5 ± 0.3	11.6 ± 0.3	9.5 ± 0.1

^a–n^ were previously detailed by Mzengereza, et al. [20]. The experimental diets fed to red seabream for 60 days formulated by replacing fish meal with camelina meal at 0 g/kg for reference diet (CM0), 205 g/kg with soybean meal (SBM20.5), 205 g/kg with camelina meal (CM20.5) and 330 g/kg camelina meal (CM33).

**Table 2:** Amino acid content (AA g/100 g diet, dry matter basis) (mean ± standard error, *n* = 3) of the experimental diets fed to red seabream for 60 days formulated by replacing fish meal with camelina meal at 0 g/kg for reference diet (CM0), 205 g/kg with soybean meal (SBM20.5), 205 g/kg with camelina meal (CM20.5) and 330 g/kg camelina meal (CM33).

Amino Acids	CM	CM0	SBM20.5	CM20.5	CM33
Essential					
Arginine	3.24	2.53	2.57	2.54	2.85
Histidine	0.91	2.02	2.12	2.37	2.21
Isoleucine	1.64	1.89	1.95	1.87	1.96
Leucine	2.67	3.64	3.18	3.61	3.52
Lysine	1.83	4.08	4.14	3.67	3.75
Methionine	0.64	1.38	1.28	1.38	1.49
Phenylalanine	1.76	2.45	2.32	2.53	2.65
Threonine	1.68	2.12	2.12	2.3	2.2
Tryptophan	0.42	0.25	0.32	0.28	0.33
Valine	1.98	2.17	2.14	2.23	1.94
Non-essential					
Taurine	nd	0.77	0.75	0.8	0.9
Aspartic acid	3.24	4.65	4.35	4.26	4.25
Glutamic acid	6.56	8.03	8.12	8.22	8.08
Serine	2.01	2.04	2.25	2.3	2.2
Proline	2.21	3.40	2.93	3.39	3.38
Glycine	2.01	2.38	2.28	2.51	2.47
Alanine	1.09	2.70	2.37	2.56	2.24
Tyrosine	1.23	2.67	2.68	2.61	2.65

Where nd = not detected. Values are means of triplicate measurements.

**Table 3:** Anti-nutritional factors content in the milled camelina meals (mean ± standard error, *n* = 3).

Anti-Nutrient Factor	g/kg
Tannin	34.2 ± 0.2
Phytic acid	40.7± 0.0
Trypsin inhibitor	20.3 ± 0.0
Protease inhibitor	12.0 ± 0.1

**Table 4:** Forward (F) and reverse (R) primers were used for growth-related mRNA quantitative real-time PCR.

Name	Primer Sequence (5′–3′)	Accession Number
*β-actin*-F	TCTGTCTGGATCGGAGGTC	JN226150.1
*β-actin*-R	AAGCATTTGCGGTGGACG	
*IGF-1*-F	TAAACCCACACCGAGTGACA	AB050670.1
*IGF-1*-R	GCGATGSSGAAAAGCTACGG	
*IGF-2*-F	CGGCAAACTAGTGATGAGCA	AB360966.1
*IGF-2*-R	CAGTGTCAAGGGGGAAGTGT	

Where: *β-actin* is the housekeeping gene according to the protocol proposed by Hossain, et al. [45]; *IGF-1*: Insulin-Like Growth Factor 1; *IGF-2*; Insulin-Like Growth Factor 2.

**Table 5:** Growth variables feed utilization markers, biometric indices, and survival obtained after 60 days feeding period in red seabream fed diets formulated by replacing fish meal with camelina meal at 0% for reference diet (CM0), 20.5 g/kg with soybean mean (SBM20.5), 20.5 g/kg with camelina meal (CM20.5) and 33 g/kg camelina meal (CM33).

Parameter	CM0	SBM20.5	CM20.5	CM33
Initial weight(g/fish)	6.5	6.5	6.4	6.4
Final weight(g/fish)	29.9 ± 0.7 ^a^	28.1 ± 0.9 ^a^	28.3 ± 0.8 ^a^	24.9 ± 0.3 ^b^
SGR ^1^	2.52 ± 0.0 ^a^	2.42 ± 0.1 ^a^	2.45 ± 0.1 ^a^	2.2 ± 0.0 ^b^
FI(g/fish/56days) ^2^	27.6 ± 0.8 ^a^	24.7 ± 1.1 ^ab^	23.6 ± 1.5 ^ab^	20.6 ± 0.9 ^bc^
FCR ^3^	1.0 ± 0.0 ^b^	1.1 ± 0.1 ^b^	1.1 ± 0.0 ^b^	1.2 ± 0.1 ^a^
BWG ^4^	353.5 ± 4.9 ^a^	327.6 ± 18.5 ^a^	334.1 ± 14.3 ^a^	275.09 ± 5.8 ^b^
HIS ^5^	1.8 ± 0.1	1.6 ± 0.1	2.0 ± 0.2	1.9 ± 0.1
SR ^6^	100± 0.0	100 ± 0.0	100 ± 0.0	100 ± 0.0
CF ^7^	1.9 ± 0.1	1.9 ± 0.0	1.8 ± 0.1	1.8 ± 0.2

Values are shown as mean ± S.E. (*n* = 3). Data with the same letters in the column are not significantly different (*p* < 0.05). ^1^ Specific growth rate (SGR %/day). ^2^ FI, Feed intake (g/fish/60 days). ^3^ FCR, feed conversion ratio. ^4^ BWG, Body Weight gain (%). ^5^ HSI, Hepatosomatic index. ^6^ SR, Survival (%). ^7^ CF, Condition factor.

**Table 6:** Proximate composition (%) of the whole-body carcass obtained after 60 days feeding period in red seabream fed diets formulated by replacing fish meal with camelina meal at 0% for reference diet (CM0), 20.5 g/kg with soybean mean (SBM20.5), 20.5 g/kg with camelina meal (CM20.5) and 33 g/kg camelina meal (CM33).

Parameter	CM0	SBM20.5	CM20.5	CM33
Crude protein	21.9 ± 0.8 ^a^	20.9 ± 0.1 ^a^	20.3 ± 1.7 ^a^	18.3 ± 0.5 ^b^
Total Lipid	16.6 ± 0.4	17.8 ± 2.4	18.1 ± 0.1	18.2 ± 0.2
Moisture	69.6 ± 0.5	68.0 ± 1.2	69.4 ± 0.3	69.1 ± 0.5
Ash	4.2 ± 0.1	5.7 ± 0.1	4.6 ± 0.1	3.2 ± 0.05

Values represent means of triplicate groups ± S.E.M., *n* = 3 means with different letters are significantly different (*p* < 0.05); means with the same letters are not significantly different (*p* > 0.05)**.**

**Table 7:** Enzyme activities (U/mg protein) in the intestine obtained after 60 days feeding period in red seabream fed diets formulated by replacing fish meal with camelina meal at 0% for reference diet (CM0), 20.5 g/kg with soybean mean (SBM20.5), 20.5 g/kg with camelina meal (CM20.5) and 33 g/kg camelina meal (CM33).

Parameters	CM0	SBM20.5	CM20.5	CM33
protease	3.0 ± 0.0 ^a^	1.2 ± 0.1 ^b^	1.2 ± 0.1 ^b^	0. 8 ± 0.0 ^c^
Pepsin	1.05 ± 0.2 ^a^	0.69 ± 0.0 ^a,b^	0.63 ± 0.0 ^a,b^	0.55 ± 0.0 ^b^
Lipase	0.33 ± 0.1	0.28 ± 0.1	0.3 ± 0.1	0.2 ± 0.0
Amylase	0.6 ± 0.1 ^a^	0.14 ±0.4 ^b^	0.37 ± 0.0 ^b^	0.28 ± 0.3 ^b^

Values represent means of triplicate groups ± S.E.M., *n* = 3 means with different letters in column are significantly different (*p* < 0.05); means with the same letters are not significantly different (*p* > 0.05).

**Table 8:** Serum biochemistry parameters obtained after 60 days feeding period in red seabream fed diets formulated by replacing fish meal with camelina meal at 0% for reference diet (CM0), 20.5 g/kg with soybean mean (SBM20.5), 20.5 g/kg with camelina meal (CM20.5) and 33 g/kg camelina meal (CM33).

Parameter	Control	SBM 20.5	CM 20.5	CM 33
Hematocrit (%)	35.5 ± 0.5 ^a^	37.5 ± 1.5 ^a^	35.5 ± 2.5 ^a^	24 ± 1 ^b^
Glucose (mg/dL)	70.3 ± 5.7 ^a^	76.7 ± 4.9 ^a^	78 ± 16.7 ^a^	108 ± 20.4 ^b^
Total bilirubin (mg/dL)	0.3 ± 0.0 ^a^	0.3 ± 0.0 ^a^	0.3 ± 0.0 ^a^	0.3 ± 0.0 ^a^
Total protein (mg/dL)	3.2 ± 0.1 ^a^	3.4 ± 0.7 ^a^	3.2 ± 0.1 ^a^	3.7 ± 0.0 ^a^
T-Cho g/dL) ^1^	178 ± 58.2 ^a^	184.3 ± 28.9 ^a^	188.3 ± 14 ^a^	225 ± 2.6 ^a^
TG (g/dL) ^2^	181 ± 9.7 ^a^	173.6 ± 8.8 ^a^	164.3 ± 10.7 ^a^	170.6 ± 12.6 ^a^
AST (IU/L) ^3^	35 ± 3.4 ^a^	36.5 ± 6.4 ^a^	31.7 ± 3.7 ^a^	26.5 ± 11.5 ^a^
ALT (IU/L) ^4^	33 ± 2.3 ^a^	18 ± 3.1 ^a^	28 ± 1.8 ^a^	10.7 ± 0.6 ^a^

Values are expressed as mean ± SE from triplicate groups (*n* = 3). Data with the same alphabets in the column are not significantly different (*p* > 0.05). ^1^ T-Cho: total cholesterol. ^2^ TG: triglyceride. ^3^ AST: Aspartate transaminase. ^4^ ALT: Alanine transaminase.

**Table 9:** Apparent digestibility coefficients (%, mean ± standard error, *n* = 3) of nutrients in red seabream fed diets formulated by replacing fish meal with camelina meal at 0% for reference diet (CM0), 20.5 g/kg with soybean mean (SBM20.5), 20.5 g/kg with camelina meal (CM20.5) and 33 g/kg camelina meal (CM33).

Parameter	CM0	SBM20.5	CM20.5	CM33
Total lipid	87.8 ± 0.7	86.8 ± 0.9	84.9 ± 0.1	84.6 ± 0.6
Dry matter	72.9 ± 2.5	72.4 ± 1.4	70.4 ± 2.5	72.6 ± 0.4
Crude protein	91.9 ± 0.5 ^a^	91.2 ± 0.3 ^a^	90.8 ± 0.2 ^a^	87.6 ± 0.3 ^b^

Values are means of triplicate groups ± S.E.M, *n* = 3. Within a row, means with different letters are significantly different (*p* < 0.05); means with the same letters are not significantly different (*p* > 0.05).

**Table 10:** Table of loading for the parameters assessed with principal component analysis. PC1 and PC2 explained the total variation as displayed.

Parameter	PC1	PC2	PC3
igf1	0.24	−0.05	0.08
igf2	0.24	-0.01	0.02
Growth	0.24	−0.02	0.13
FCR	−0.24	0.04	−0.12
FI	0.23	0.14	0.06
SGR	0.21	−0.21	0.44
ADC CP	0.23	−0.12	−0.27
ADC TL	0.20	0.35	−0.35
Protease	0.20	0.4	0.34
Pepsin	0.20	0.41	0.20
Lipase	0.24	−0.05	0.07
d-ROMs	−0.23	0.16	0.20
BAP	0.23	0.13	0.15
SOD	0.20	0.40	−0.33
TBARs	−0.24	−0.08	0.11
Catalase	0.23	−0.11	−0.35
Lysozyme	0.23	−0.14	0.12
NBT	0.22	−0.27	0.05
Hematocrit	0.21	−0.30	−0.28
Peroxidase	0.23	−0.24	0.10

## Data Availability

The datasets generated during and analysed during the current study are available from the corresponding author on reasonable request.

## References

[1] Wei M., Anderson D.M., Zhang Z., Colombo S.M. (2020). High-oil residue camelina meal, a viable source of protein at low levels in diets for juvenile salmonids. Aquac. Nutr..

[2] Ido A., Iwai T., Ito K., Ohta T., Mizushima T., Kishida T., Miura C., Miura T. (2015). Dietary effects of housefly (*Musca Domestica*) (Diptera: Muscidae) pupae on the growth performance and the resistance against bacterial pathogen in red sea bream (*Pagrus major*) (Perciformes: Sparidae). Appl. Entomol. Zool..

[3] Bianchi M.C.G., Chopin F., Farme T., Franz N., Fuentevilla C., Garibaldi L., Laurenti A.L.G. (2020). FAO: The State of World Fisheries and Aquaculture.

[4] Dawood M.A.O. (2021). Nutritional immunity of fish intestines: Important insights for sustainable aquaculture. Rev. Aquac..

[5] Cardinaletti G., Randazzo B., Messina M., Zarantoniello M., Giorgini E., Zambelli A., Bruni L., Parisi G., Olivotto I., Tulli F. (2019). Effects of graded dietary inclusion level of full-fat *Hermetic illucens* prepupae meal in practical diets for rainbow trout (*Oncorhynchus mykiss*). Animals.

[6] Biswas A., Araki H., Sakata T., Nakamori T., Kato K., Takii K. (2017). Fish meal replacement by soy protein from soymilk in the diets of red sea bream (*Pagrus major*). Aquac. Nutr..

[7] Dossou S., Koshio S., Ishikawa M., Yokoyama S., Dawood M.A.O., El Basuini M.F., El-Hais A.M., Olivier A. (2018). Effect of partial replacement of fish meal by fermented rapeseed meal on growth, immune response and oxidative condition of red sea bream juvenile, *Pagrus major*. Aquaculture.

[8] Seong T., Matsutani H., Haga Y., Kitajima R., Satoh S. (2019). First step of non-fish meal, non-fish oil diet development for red seabream, (*Pagrus major*), with plant protein sources and microalgae *Schizochytrium* sp.. Aquac. Res..

[9] Seong T., Kitajima R., Haga Y., Satoh S. (2020). Non-fish meal, non-fish oil diet development for red sea bream, *Pagrus major*, with plant protein and graded levels of *Schizochytrium* sp.: Effect on growth and fatty acid composition. Aquac. Nutr..

[10] Espe M., Lemme A., Petri A., El-Mowafi A. (2006). Can Atlantic salmon (*Salmo salar*) grow on diets devoid of fish meal?. Aquaculture.

[11] Jiang D., Zheng J., Dan Z., Tang Z., Ai Q., Mai K. (2019). Effects of five compound attractants in high plant-based diets on feed intake and growth performance of juvenile turbot (*Scophthalmus maximus* L.). Aquac. Res..

[12] Dawood M.A.O., Koshio S. (2020). Application of fermentation strategy in aquafeed for sustainable aquaculture. Rev. Aquac..

[13] Mazumder A., Dwivedi A., Du Plessis J. (2016). Sinigrin and its therapeutic benefits. Molecules.

[14] Glencross B.D., Baily J., Berntsen M.H.G., Hardy R., MacKenzie S., Tocher D.R. (2020). Risk assessment of the use of alternative animal and plant raw material resources in aquaculture feeds. Rev. Aquac..

[15] Mudalkar S., Golla R., Ghatty S., Reddy A.R. (2014). De novo transcriptome analysis of an imminent biofuel crop, *Camelina sativa* l. Using Illumina gaiix sequencing platform and identification of SSR markers. Plant Mol. Biol..

[16] Onyilagha J., Bala A., Hallett R., Gruber M., Soroka J., Westcott N. (2003). Leaf flavonoids of the cruciferous species, *Camelina sativa*, Crambe spp., Thlaspi arvense and several other genera of the family Brassicaceae. Biochem. Syst. Ecol..

[17] Hixson S.M., Parrish C.C. (2014). Substitution of fish oil with camelina oil and inclusion of camelina meal in diets fed to Atlantic cod (*Gadus morhua*) and their effects on growth, tissue lipid classes, and fatty acids1. J. Anim. Sci..

[18] Betancor M.B., Li K., Bucerzan V.S., Sprague M., Sayanova O., Usher S., Han L., Norambuena F., Torrissen O., Napier J.A. (2018). Oil from transgenic *camelina sativa* containing over 25 % n-3 long-chain pufa as the major lipid source in feed for Atlantic salmon (*Salmo salar*). Br. J. Nutr..

[19] Toyes-Vargas E.A., Parrish C.C., Viana M.T., Carreón-Palau L., Magallón-Servín P., Magallón-Barajas F.J. (2020). Replacement of fish oil with camelina (*Camelina sativa*) oil in diets for juvenile tilapia (var. Gift *Oreochromis niloticus*) and its effect on growth, feed utilization and muscle lipid composition. Aquaculture.

[20] Mzengereza K., Ishikawa M., Koshio S., Yokoyama S., Yukun Z., Shadrack R.S., Seo S., Duy Khoa T.N., Moss A., Dossou S. (2021). Effect of substituting fish oil with camelina oil on growth performance, fatty acid profile, digestibility, liver histology, and antioxidative status of red seabream (*Pagrus major*). Animals.

[21] Bullerwell C.N., Collins S.A., Lall S.P., Anderson D.M. (2016). Growth performance, proximate and histological analysis of rainbow trout fed diets containing *Camelina Sativa* seeds, meal (high-oil and solvent-extracted) and oil. Aquaculture.

[22] Hixson S.M., Parrish C.C., Wells J.S., Winkowski E.M., Anderson D.M., Bullerwell C.N. (2016). Inclusion of camelina meal as a protein source in diets for farmed salmonids. Aquac. Nutr..

[23] Agency C.F.I. (2017). The Biology of *Aamelina sativa* (L.) Crantz (Camelina). Directive 94–08 (dir94-08), Assessment Criteria for Determining Environmental Safety of Plant with Novel Traits. http://www.Inspection.Gc.Ca/plants/plants-withnovel-traits/applicants/directive-94-08/biology-documents/camelina-Sativa-l-/eng/1330971423348/1330971509470.

[24] Matthäs B. (1997). Antinutritive compounds in different oilseeds. Lipid/Fett.

[25] Dossou S., Dawood M.A.O., Zaineldin A.I., Abouelsaad I.A., Mzengereza K., Shadrack R.S., Zhang Y., El-Sharnouby M., Ahmed H.A., El Basuini M.F. (2021). Dynamical hybrid system for optimizing and controlling efficacy of plant-based protein in aquafeeds. Complexity.

[26] Olivotto I., Mosconi G., Maradonna F., Cardinali M., Carnevali O. (2002). *Diplodus sargus* interrenal–pituitary response: Chemical communication in stressed fish. Gen. Comp. Endocrinol..

[27] Panserat S., Kaushik S.J. (2010). Regulation of gene expression by nutritional factors in fish. Aquac. Res..

[28] Picha M.E., Turano M.J., Beckman B.R., Borski R.J. (2008). Endocrine biomarkers of growth and applications to aquaculture: A minireview of growth hormone, insulin-like growth factor (IGF)-i, and IGF-binding proteins as potential growth indicators in fish. N. Am. J. Aquac..

[29] Duan C. (1998). Nutritional and developmental regulation of insulin-like growth factors in fish. J. Nutr..

[30] Luo Y., Ai Q., Mai K., Zhang W., Xu W., Zhang Y. (2012). Effects of dietary rapeseed meal on growth performance, digestion and protein metabolism in relation to gene expression of juvenile cobia (*Rachycentron canadum*). Aquaculture.

[31] NRC (2011). Nutrient requirements of fish and shrimp. Animal Nutrition Series, National Research Council of the National Academies.

[32] AOAC (2012). Official Methods of Analysis of Aoac International.

[33] Bligh E.G., Dyer W.J. (1959). A rapid method of total lipid extraction and purification. Can. J. Biochem. Physiol..

[34] Teshima S.-I., Kanazawa A., Yamashita M. (1986). Dietary value of several proteins and supplemental amino acids for larvae of the prawn *Penaeus japonicus*. Aquaculture.

[35] Kader M.A., Koshio S., Ishikawa M., Yokoyama S., Bulbul M. (2010). Supplemental effects of some crude ingredients in improving nutritive values of low fishmeal diets for red sea bream, *Pagrus major*. Aquaculture.

[36] Qing P.L., Xing W.K. (1997). The experimental studies on activities of digestive enzyme in the larvae *Penaeus chinensis*. J. Fish. China.

[37] Natalia Y., Hashim R., Ali A., Chong A. (2004). Characterization of digestive enzymes in a carnivorous ornamental fish, the Asian bony tongue *Scleropages formosus* (osteoglossidae). Aquaculture.

[38] Roberts I.M. (1985). Hydrolysis of 4-methylumbelliferyl butyrate: A convenient and sensitive fluorescent assay for lipase activity. Lipids.

[39] Morishita K., Matsunari H., Furuta H., Rønnestad I., Oku H., Yamamoto T. (2018). Effects of dietary soybean meal on the digestive physiology of red sea bream *Pagrus major*. Aquaculture.

[40] Salinas I., Abelli L., Bertoni F., Picchietti S., Roque A., Furones D., Cuesta A., Meseguer J., Esteban M.Á. (2008). Monospecies and multispecies probiotic formulations produce different systemic and local immunostimulatory effects in the gilthead seabream (*Sparus aurata* l.). Fish Shellfish Immunol..

[41] Lygren B., Sevier H., Hjeltness B., Waagbø R. (1999). Examination of the immunomodulatory properties and the effect on disease resistance of dietary bovine lactoferrin and vitamin c fed to Atlantic salmon (*Salmo salar*) for a short-term period. Fish Shellfish Immunol..

[42] Anderson D., Siwicki A. (1995). Basic Hematology and Serology for Fish Health Programs.

[43] Moe Y.Y., Koshio S., Teshima S.I., Ishikawa M., Matsunaga Y., Panganiban A. (2004). Effect of vitamin c derivatives on the performance of larval kuruma shrimp, *Marsupenaeus japonicus*. Aquaculture.

[44] Furukawa A. (1966). On the acid digestion method for the determination of chromic oxide as an index substance in the study of digestibility of fish feed. Nippon. Suisan Gakkaishi.

[45] Hossain M.S., Koshio S., Ishikawa M., Yokoyama S., Sony N.M., Dawood M.A.O., Kader M.A., Bulbul M., Fujieda T. (2016). Efficacy of nucleotide related products on growth, blood chemistry, oxidative stress and growth factor gene expression of juvenile red sea bream, *Pagrus major*. Aquaculture.

[46] Hammer Ø., Harper D.A., Ryan P. (2001). Paleontological statistics software package for education and data analysis. Paleontol. Electron..

[47] Dawood M.A.O., Eweedah N.M., Khalafalla M.M., Khalid A., Astley A.E., Fadl S.E., Amin A.A., Paray B.A., Ahmed H.A. (2020). *Saccharomyces cerevisiae* increases the acceptability of Nile tilapia (*Oreochromis niloticus*) to date palm seed meal. Aquac. Rep..

[48] Brown T.D., Hori T.S., Xue X., Ye C.L., Anderson D.M., Rise M.L. (2016). Functional genomic analysis of the impact of camelina (*camelina sativa*) meal on Atlantic salmon (*Salmo salar*) distal intestine gene expression and physiology. Mar. Biotechnol..

[49] Pan X., Xie W., Caldwel C., Anderson D. (2011). Growth performance and carcass composition of rainbow trout (*Oncorhynchus mykiss*) fed practical diets containing graded levels of high fat residue camelina meal. Can. J. Anim. Sci..

[50] Matthäus B., Zubr J. (2000). Variability of specific components in *Camelina sativa* oilseed cakes. Ind. Crop. Prod..

[51] Adeoye A.A., Jaramillo-Torres A., Fox S.W., Merrifield D.L., Davies S.J. (2016). Supplementation of formulated diets for tilapia (*Oreochromis niloticus*) with selected exogenous enzymes: Overall performance and effects on intestinal histology and microbiota. Anim. Feed. Sci. Technol..

[52] Hassaan M.S., Goda A.M.A.S., Kumar V. (2017). Evaluation of nutritive value of fermented de-oiled physic nut, jatropha curcas, seed meal for Nile tilapia *Oreochromis niloticus* fingerlings. Aquac. Nutr..

[53] Refstie S., Storebakken T., Roem A.J. (1998). Feed consumption and conversion in Atlantic salmon (*Salmo salar*) fed diets with fish meal, extracted soybean meal or soybean meal with reduced content of oligosaccharides, trypsin inhibitors, lectins and soya antigens. Aquaculture.

[54] Nagel F., Appel T., Rohde C., Kroeckel S., Schulz C. (2017). Blue mussel protein concentrate versus prime fish meal protein as a dietary attractant for turbot (*Psetta maxima* L.) given rapeseed protein-based diets. Aquac. Res. Dev. S.

[55] Kokou F., Fountoulaki E. (2018). Aquaculture waste production associated with antinutrient presence in common fish feed plant ingredients. Aquaculture.

[56] Eppler E., Berishvili G., Mazel P., Callers A., Hwang G., Maclean N., Reinecke M. (2010). Distinct organ-specific up-and down-regulation of igf-i and igf-ii mRNA in various organs of a gh-overexpressing transgenic Nile tilapia. Transgenic Res..

[57] Jiménez-Amilburu V., Salmerón C., Codina M., Navarro I., Capilla E., Gutiérrez J. (2013). Insulin-like growth factors effects on the expression of myogenic regulatory factors in gilthead sea bream muscle cells. Gen. Comp. Endocrinol..

[58] Bu X.-Y., Wang Y.-Y., Chen F.-Y., Tang B.-B., Luo C.-Z., Wang Y., Ge X.-P., Yang Y.-H. (2018). An evaluation of replacing fishmeal with rapeseed meal in the diet of *Pseudobagrus ussuriensis*: Growth, feed utilization, non-specific immunity, and growth-related gene expression. J. World Aquac. Soc..

[59] Bu X., Lian X., Zhang Y., Chen F., Tang B., Ge X., Yang Y. (2018). Effects of replacing fish meal with corn gluten meal on growth, feed utilization, nitrogen and phosphorus excretion and igf-i gene expression of juvenile *Pseudobagrus ussuriensis*. Aquac. Res..

[60] Hevrøy E.M., Ezpeleta C., Shimizu M., Lanzén A., Kaiya H., Espe M., Olsvik P.A. (2011). Effects of short-term starvation on ghrelin, gh-igf system, and IGF-binding proteins in Atlantic salmon. Fish Physiol. Biochem..

[61] Kumar V., Makkar H.P.S., Becker K. (2011). Detoxified *Jatropha curcas* kernel meal as a dietary protein source: Growth performance, nutrient utilization and digestive enzymes in common carp (*Cyprinus Carpio* L.) fingerlings. Aquac. Nutr..

[62] Gómez-Requeni P., Calduch-Giner J., Vega-Rubín de Celis S., Médale F., Kaushik S.J., Pérez-Sánchez J. (2005). Regulation of the somatotropic axis by dietary factors in rainbow trout (*Oncorhynchus*
*mykiss*). Br. J. Nutr..

[63] Eslamloo K., Falahatkar B., Yokoyama S. (2012). Effects of dietary bovine lactoferrin on growth, physiological performance, iron metabolism and non-specific immune responses of Siberian sturgeon *Acipenser baeri*. Fish Shellfish Immunol..

[64] Lin S., Luo L. (2011). Effects of different levels of soybean meal inclusion in replacement for fish meal on growth, digestive enzymes and transaminase activities in practical diets for juvenile tilapia, *Oreochromis niloticus* × *O. aureus*. Anim. Feed. Sci. Technol..

[65] Shen J., Liu H., Tan B., Dong X., Yang Q., Chi S., Zhang S. (2020). Effects of replacement of fishmeal with cottonseed protein concentrate on the growth, intestinal microflora, haematological and antioxidant indices of juvenile golden pompano (*Trachinotus ovatus*). Aquac. Nutr..

[66] Piccinetti C.C., Grasso L., Maradonna F., Radaelli G., Ballarin C., Chemello G., Evjemo J.O., Carnevali O., Olivotto I. (2017). Growth and stress factors in Ballan wrasse (*Labrus bergylta*) larval development. Aquac. Res..

[67] Dawood M.A.O., Koshio S., Fadl S.E., Ahmed H.A., El Astley A., Abdel-Daim M.M., Alkahtani S. (2020). The modulatory effect of mannan oligosaccharide on oxidative status, selected immune parameters and tolerance against low salinity stress in red sea bream (*Pagrus major*). Aquac. Rep..

[68] KUBİLAY A., ULUKÖY G. (2002). The effects of acute stress on rainbow trout (*Oncorhynchus mykiss*). Turk. J. Zool..

[69] Tovar-Ramírez D., Mazurais D., Gatesoupe J.F., Quazuguel P., Cahu C.L., Zambonino-Infante J.L. (2010). Dietary probiotic live yeast modulates antioxidant enzyme activities and gene expression of sea bass (*Dicentrarchus labrax*) larvae. Aquaculture.

